# Missense mutation of a class B heat shock factor is responsible for the tomato *bushy root-2* phenotype

**DOI:** 10.1186/s43897-022-00025-0

**Published:** 2022-02-08

**Authors:** Zoltan Kevei, Silva Demetryus Silva Ferreira, Cristina Maria Perez Casenave, Tomasz Kurowski, Fady Mohareb, Daniel Rickett, Chris Stain, Andrew J. Thompson

**Affiliations:** 1 Cranfield Soil and AgriFood Institute, College Road, Cranfield University, Bedfordshire, MK43 0AL UK; 2 Syngenta Crop Protection, Jealott’s Hill International Research Centre, Bracknell, Berkshire, RG42 6EY UK

**Keywords:** *Bushy root-2*, Genetic mapping, *HsfB4*, Root knot nematode resistance, *SCHIZORIZA*, Tomato

## Abstract

The *bushy root-2* (*brt-2*) tomato mutant has twisting roots, and slower plant development. Here we used whole genome resequencing and genetic mapping to show that *brt-2* is caused by a serine to cysteine (S75C) substitution in the DNA binding domain (DBD) of a heat shock factor class B (HsfB) encoded by *SolycHsfB4a*. This gene is orthologous to the Arabidopsis *SCHIZORIZA* gene, also known as *AtHsfB4*. The *brt-2* phenotype is very similar to *Arabidopsis* lines in which the function of *AtHsfB4* is altered: a proliferation of lateral root cap and root meristematic tissues, and a tendency for lateral root cap cells to easily separate. The *brt-2* S75C mutation is unusual because all other reported amino acid substitutions in the highly conserved DBD of eukaryotic heat shock factors are dominant negative mutations, but *brt-2* is recessive. We further show through reciprocal grafting that *brt-2* exerts its effects predominantly through the root genotype even through *BRT-2* is expressed at similar levels in both root and shoot meristems. Since *AtHsfB4* is induced by root knot nematodes (RKN), and loss-of-function mutants of this gene are resistant to RKNs, *BRT-2* could be a target gene for RKN resistance, an important trait in tomato rootstock breeding.

**Gene & accession numbers**

*SolycHsfB4a* - *Solyc04g078770*.

**Supplementary Information:**

The online version contains supplementary material available at 10.1186/s43897-022-00025-0.

## Core

The *bushy root-2* (*brt-2*) tomato mutant has twisting roots and its genetic mapping revealed that the phenotype is caused by a serine to cysteine substitution in the DNA binding domain of a class B heat shock factor protein encoded by *SolycHsfB4a*. Since *AtHsfB4* is induced by root knot nematodes (RKN), and its loss-of-function mutants are resistant to RKNs, *BRT-2* could be a target gene for RKN resistance, an important trait in tomato rootstock breeding.

## Introduction

Root architecture is plastic and important for water and mineral absorption, anchorage and storage (Nibau et al., 2008). Changes in root function and architecture have resulted in enhancements for crop production (Hammer et al., 2009; Siddiqui et al., 2021), and much has been achieved to understand the genetic regulation of root system architecture and development, particularly in *Arabidopsis* (Motte et al., 2019). Breeding for improved root systems is of great interest for grafted vegetable production where elite scion genotypes with favourable aboveground traits are grafted onto rootstocks, especially in the solanaceous crops tomato, pepper and eggplant (Thompson et al., 2017). When choosing rootstocks, the foremost interests are the overall yield, resistance against biotic and abiotic stresses and improved resource use efficiency (Martínez-Andújar et al., 2020). Rootstocks have been selected to challenge extreme conditions, such as low nutrient availability (Schwarz et al., 2013), hydric stress (Sánchez-Rodríguez et al., 2012), high salinity (Santa-Cruz et al., 2002) and pest control (Gregory et al., 2013; Gálvez et al., 2019). Breeding for tomato rootstocks requires an understanding of the genetic variation for root traits and the available germplasm resources that can be applied to rootstock breeding (Pico et al., 2017).

In tomato there have been relatively few investigations linking root traits to loci and genes. Although a recent study developed methods to rapidly identify seedling root mutants via EMS mutagenesis in the dwarf tomato cultivar Micro-Tom (Alaguero-Cordovilla and Belén Sánchez-García, 2021), the mutant collection of the C.M. Rick Tomato Genetics Resource Centre (TGRC, David, California), already includes 15 monogenic tomato mutants with distinctive root phenotypes (Table 1). For the further understanding of molecular processes impacting root development and rootstock characteristics in tomato, we have investigated several of these root mutants. One of these mutants, *bushy root-2* (*brt-2*), possess a twisting tap root, and lateral roots were reported to arise at high density giving a bushy appearance (Voland and Zobel, 1988). The lateral and basal roots also curl and twist, and the shoot growth of *brt-2* is relatively slower than other tomato lines. The *brt-2* mutant was previously crossed with a series of classical tomato mutants and was found to be linked to four mutant loci: *clausa*, *fulgens*, *entire* and *divergens*, indicating that the *brt-2* locus maps at 40–45 cM on chromosome 4 (Voland and Zobel, 1988). The *entire* locus showed the closest linkage with *brt-2* and was subsequently identified as a single-base deletion in the *SlIAA9* gene (*Solyc04g076850*), a transcriptional repressor of auxin signalling impacting leaf morphogenesis and fruit development (Zhang et al., 2007).

**Table 1:** Monogenic TGRC mutant lines exhibiting significant root phenotype

Mutant	Overall phenotype	Predominantly root phenotype?	Locus?	Reference
*aerial roots (aer)*	Adventitious roots on stem from soil level to considerable height above	Yes	unknown	(Philouze, 1971)
*aerial roots-2 (aer-2)*	Abundance of root initials along the stems.	Yes	unknown	(Kerr, 1982)
*baby lea syndrome (bls)*	Anthocyaninless; restricted root system; short internodes, leaves, and trusses	No	chr 3	(Clayberg et al., 1966)
*bushy root (brt)*	Radical branches early; radical and root profusely branched; root tips twisting upwards, not dense growth.	Yes	chr 12	(Zobel, 1972)
*bushy root 2 (brt-2)*	Severely stunted growth (1/20) dense bushy growth of twisted roots	Yes	chr 4	(Voland and Zobel, 1988)
*cottony root (crt)*	Overgrown root hairs with cottony appearance	Yes	unknown	(Hochmuth, 1985)
*decumbens (dec)*	Lax and decumbent habit; early fruiting	No	unknown	(Stubbe, 1959)
*diageotropica (dgt)*	Plant habit prostrate due to reduced gravitropic response; growth retarded; stems and leaves droopy; cotyledons concave. Roots grow horizontally rather than downwards; no lateral root formation.	No	*Solyc01g111170*	(Oh et al., 2006)
*dwarf root (drt)*	Reduced hypocotyl and internodes, compact root phenotype	Yes	chr 2	(Voland and Zobel, 1988)
*lembiformis (le)*	Prostrate, smaller plant, proportionately reduced; keeled or involuted yellowish pinnae, ventrally purplish.	No	unknown	(Stubbe, 1964)
*ridged (ri)*	Ridged leaves; retarded growth of shoots and roots.	No	chr 6	(Lindstrom, 1933)
*Root suppressed (Rs)*	Greatly restricted or no root development	Yes	chr 4	(Yu, S. -a. and Yeager, A.F., 1960)
*umbrosa (um)*	Mature leaves darker green, wilted appearance; later growth stunted; reduced root growth	No	chr 1	(Stubbe, 1958)
*wilty dwarf (wd)*	Greyish-green, droopy leaves; stunted plants; leaves droop when drought stressed	Yes	chr 9	(Rick and Khush, 1961)

Here we aim to identify the causative gene for *brt-2* phenotype. Next generation sequencing (NGS) technologies have made studies linking phenotype to genotype faster and cheaper. The tomato reference genome based on cv. Heinz 1706 (Sato et al., 2012) is extensively used for SNP identification between different genomes. SNP and InDel polymorphisms are described in over 500 accessions of tomato (Aflitos et al., 2014; Kim et al., 2014; Lin et al., 2014), and a pan genome (Gao et al., 2019) and genome-wide structural variants (Alonge et al., 2020) are also described. These data sets and low-cost whole genome NGS in tomato make fine genetic mapping of mutants highly amenable. In this study we identify an excellent candidate gene for *brt-2* through sequencing and fine mapping, and use grafting and microscopic analyses to further define the *brt-2* phenotype.

## Results

### Observations of the *brt-2* phenotype

**Fig. 1:**
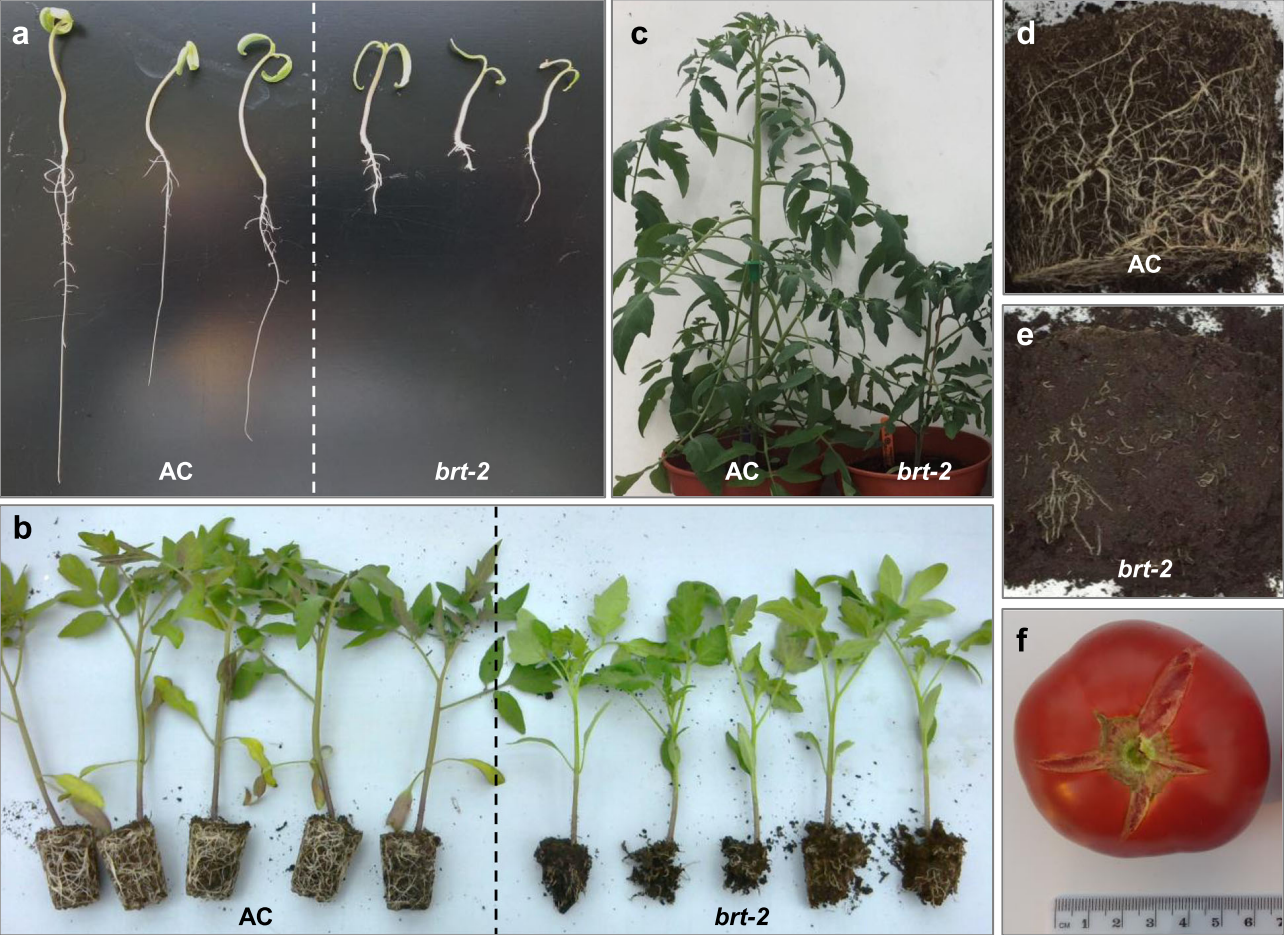
The *brt-2* phenotype. **a** comparison of seedling growth of *brt-2* and AC. **b** comparison of 4 weeks old AC and *brt-2* lines. **c** 9 weeks old AC and *brt-2* plants possess different shoot size. **d** 9 weeks old AC produces significantly more roots than *brt-2* plants **e** in 10 L pots. **f** The *brt-2* tomatoes usually possess cracks

The TGRC entry describes the *bushy root-2* tomato line (LA3206) as a spontaneous mutant within an unknown genetic background showing severely stunted growth and possessing dense, bushy, twisted roots. Compared to AC, *brt-2* roots exhibited a strongly decreased growth, which is already noticeable at the cotyledon stage (Fig. 1a). The four-week-old *brt-2* plants also showed decreased shoot development and curly roots with visibly reduced root length density, generally lacking finer lateral roots (Fig. 1b). The established *brt-2* plants possessed shorter shoot (Fig. 1c) and extremely reduced root system compared to AC with striking difference (Fig. 1d and e). The young leaves of *brt-2* were epinastic (laminar and petiole tending to curve downwards) and the leaves were observed to have a tendency to wilt in the glasshouse in well-watered conditions when evaporative demand was high (high temperature, low relative humidity and high incident solar radiation). Despite the decreased root system and wilted shoot, the *brt-2* plant produced 7–8 cm sized fruit with high seed set; however they had a high tendency for radial cracking (Fig. 1f). When the mutant line was grown in an aeroponic system, the size difference compared to AC was even more stark (data not shown).

### Reciprocal grafting of AC and *brt-2*

**Fig. 2:**
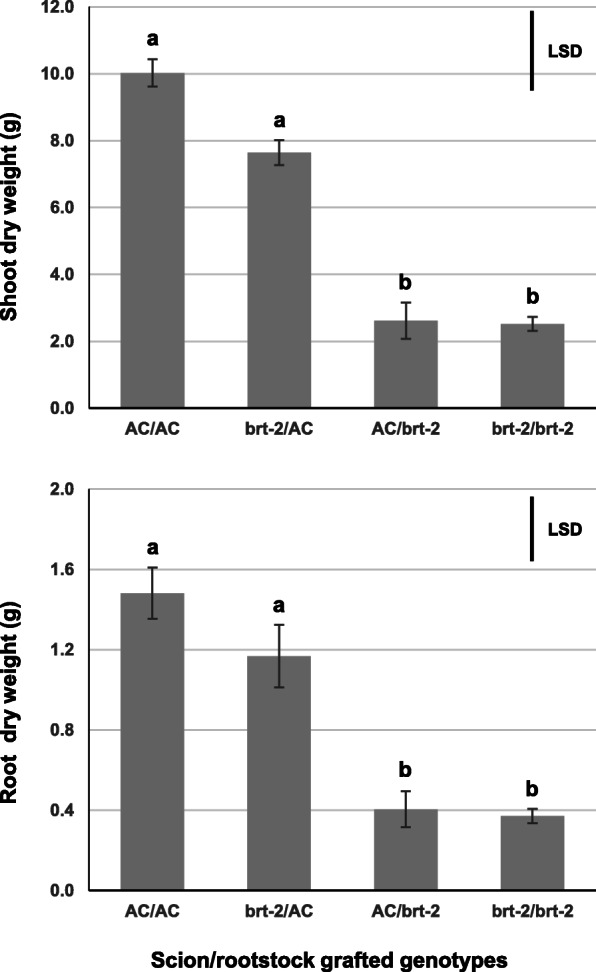
Reciprocal grafting of AC and *brt-2* lines. 9-week-old pot-grown plants were used for shoot (upper panel) and root (lower panel) dry weight measurements (Table S1). Standard errors are indicated, Least Significant Difference (LSD) of the one-way ANOVA are marked (*p* < 0.05)

In order to investigate the tissue specific impacts of the *brt-2* locus on the whole plant phenotype, we made reciprocal grafting with AC in the four possible combinations including self-grafted genotypes (shoot/root): AC/*brt-2*, *brt-2*/AC, AC/AC and *brt-2*/*brt-*2. After 9 weeks the shoot and root dry weights (DW) were obtained (Fig. 2; Table S1). Self-grafted AC/AC plants were significantly larger than *brt*/*brt* plants, confirming the negative effect of the *brt-2* mutation observed (Fig. 1) and providing quantification of the difference; shoot DW and root DW were both 3.4-fold greater in AC/AC vs *brt-2*/*brt-2*. The AC shoot in AC/*brt-2* grafts was much smaller than in AC/AC grafts, indicating that the mutant rootstocks impaired shoot growth. In contrast, both AC and *brt-2* scions showed similar growth when grafted onto AC rootstocks (although the *brt-2* shoot DW was ~ 24% less than AC shoot growth, this was not statistically significant). The dry weight of *brt-2* shoots was increased 3-fold by using AC rather than *brt-2* as rootstock, but the AC scion was not able to increase the *brt-2* root mass in the AC/*brt-2* graft compared to *brt-2*/*brt-2*. These data indicate that the effects of the *brt-2* mutation are only expressed when the mutation is present in the root; a shoot containing the *brt-2* mutation grows normally if its rootstock carries the wild type (WT) allele (*brt-2*^*+*^).

### Genetic mapping of the *brt-2* locus

The *brt-2* mutant line was crossed with AC to create an F_2_ population that segregated with WT (219 lines) and *brt-2* (69 lines) phenotype with an approximate 3:1 ratio (Fig. S1). This classifies *brt-2* as a monogenic, recessive trait confirming the *brt-2* locus description of the TGRC database.

**Fig. 3:**
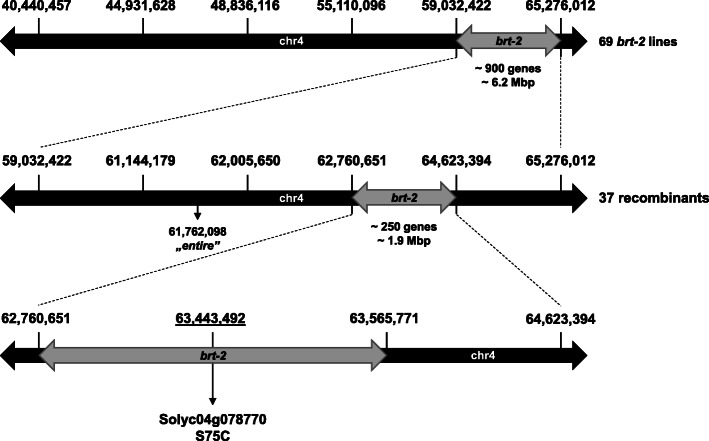
Schematic summary of the genetic mapping process of *brt-2* locus. The SL2.50 positions (bp) of SNP/InDel used for KASP marker design are indicated. The position of “*entire*” mutant (cytosine deletion) in the closely linked *SLAA9* gene is marked. The position of *brt-2* causative mutation is underlined and resulting amino acid change is presented

To identify polymorphisms for fine mapping we initially used genotype-by-sequencing (Elshire et al., 2011), but since very few polymorphisms were obtained within this cross, whole genome resequencing was performed. Based on this data we designed and tested KASP markers for six SNPs polymorphic between AC and *brt-2* lines between ~ 40 and 65 Mbp on chromosome 4 (Table S2; Fig. 3). Of the 69 F_2_ plants with the *brt-2* phenotype, 37 were recombinant allowing *brt-2* to be mapped between 59,032,422 and 65,276,012 bp (reference SL2.50), containing approximately 900 genes. Four more KASP markers were designed and scored within the recombinants and the mapping region was reduced to 1.9 Mbp between 62,760,651 and 64,623,394 bp (Fig. 3).

### *Solyc04g078770* is the only candidate gene for *brt-2*

This 1.9 Mbp region contains approximately 250 genes, however, the NGS analyses revealed only ten SNP/InDel variations in the *brt-2* parental sequence compared to AC (Table 2). Five of the ten variations were located in intergenic regions, distantly from genes. Two of the gene-related variations were within intron sequences (*Solyc04g078000*, *Solyc04g080010*), and one was in a putative promoter sequence (*Solyc04g080020*). The remaining two SNPs caused amino acid modifications, a serine-cysteine change in *Solyc04g078770* and a proline-histidine conversion in *Solyc04g080120* (Table 2). When all ten sequence variations are compared to the 150 Tomato Resequencing Project (Aflitos et al., 2014) and Tomato 360 Resequencing Project (Lin et al., 2014), only two of these changes were unique for the *brt-2* line. One is an intergenic SNP at 62,760,651 bp, already excluded from the mapping interval by the marker at this position (Fig. 3). The other is the A/T change causing a Ser to Cys (S75C) substitution in the 1st exon of *Solyc04g078770* at 63,443,492 bp. With a further two KASP markers (Fig. 3; markers at 63,443,492 and 63,565,771) we tested the linkage of the *Solyc04g078770* mutation to the *brt-2* phenotype in the 69 recombinant F_2_ lines (Table 2). The A/T change in *Solyc04g078770* was the only polymorphism that showed 100% linkage with the *brt-2* phenotype, thus formally defining the mapping interval to 0.8 Mbp (62.76 Mbp to 63.57 Mbp), a region only containing three polymorphisms, two of which could be excluded because they were common to other tomato accessions lacking the *brt-2* phenotype. So, finally the 63,443,492 SNP in *Solyc04g078770* was the only unique polymorphism in the mapping interval, it co-segregated with the *brt-2* phenotype, and caused an amino acid change; this evidence indicates very strongly that the mutation in *Solyc04g078770* causes of the *brt-2* phenotype.

**Table 2:** Sequence variations in *brt-2* compared to other tomato species in the 1.9 Mbp mapping region

Position in SL2.50	SNP/InDel	region	gene	AA change	unique variation	KASP marker	linkage to ***brt-2***
62,760,651	G/A	intergenic	n/a	n/a	yes	yes	90%
62,869,990	G/A	intron	*Solyc04g078000*	n/a	no	no	n/a
63,083,160	G/GC	intergenic	n/a	n/a	no	no	n/a
**63,443,492**	**A/T**	**exon**	***Solyc04g078770***	**Ser to Cys**	**yes**	**yes**	**100%**
63,565,771	G/T	intergenic	n/a	n/a	no	yes	99%
64,082,367	T/A	intergenic	n/a	n/a	no	no	n/a
64,309,622	T/A	intron	*Solyc04g080010*	n/a	no	no	n/a
64,313,709	T/−	promoter	*Solyc04g080020*	n/a	no	no	n/a
64,381,246	G/T	exon	*Solyc04g080120*	Pro to His	no	no	n/a
64,623,394	A/G	intergenic	n/a	n/a	no	yes	84%

Unique variants are not present in the re-sequenced lines of SGN database. KASP marker accessibility and the linkage to the *brt-*2 phenotype are marked. The proposed causative gene for *brt-2* is in bold

### The S75C mutation is predicted to have a large impact on the “class B heat shock factor” protein function

**Fig. 4:**
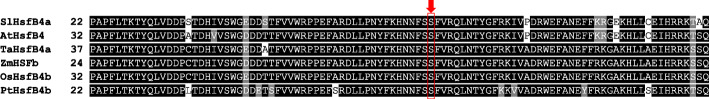
BoxShade presentation of the highly conserved HSF-DNA binding domains of SolycHsfB4a of various plant species. The serine (marked in red) is replaced by cysteine in the *brt-2* line. At, *Arabidopsis thaliana*; Ta, *Triticum aestivum*; Zm, *Zea mays*; Os, *Oryza sativa*; Pt, *Populus trichocarpa*

*Solyc04g078770,* also known as *SolycHsfB4a* (Berz et al., 2019) codes for a heat stress transcription factor, HsfB4a, which is the orthologue of the *Arabidopsis* SCHIZORIZA (SCZ) protein (ten Hove et al., 2010). *SCZ* is a member of a large gene family containing a highly conserved Hsf DNA-binding domain (DBD) motif in the first part of the coded protein (Ahn et al., 2001). We compared the DBD domain of SolycHsfB4a with orthologue proteins in other plant species to investigate the impact of the amino acid change in the *brt-2* line (Fig. 4). The serine/cysteine replacement occurs in an extremely conserved part of the DBD domain, therefore we tested if the mutation has potential effects on the protein function. In the PROVEAN software, the S75C mutation scored − 4.858. PROVEAN scores < − 2.5 denote a potential functional shift (Choi et al., 2012) and so S75C is indeed predicted to cause a critical change in SolycHsfB4a function.

### Microscopic analyses of *brt-2* roots

**Fig. 5:**
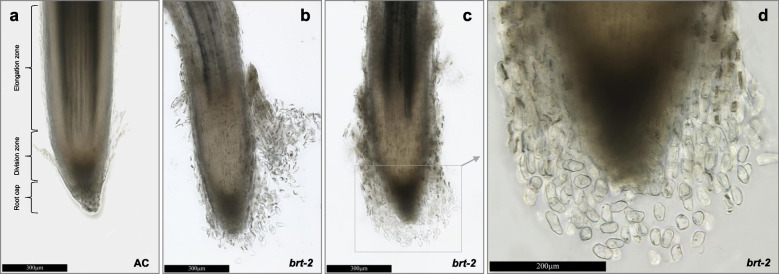
Composite light microscopy images of AC and *brt-2* mutant root tips. Roots from seven-day-old plant of AC (**a**) and brt-2 (**b-d**) were used for imaging, scale bars are shown

The *A. thaliana SCHIZORIZA* gene is involved in root development (Mylona et al., 2002), therefore we investigated related root phenotypes in the *brt-2* mutant. An image analyses showed that, compared to AC roots, the *brt-2* line possesses a drastically increased root cap and cell division zone with a large number of extra cells especially in the division zone (Fig. 5). The presence of this extra tissue is associated with the separation from the root tip of lateral root cap cells and multicellular fragments (Fig. 5b-c); there are also numerous cells that have separated from the columella tip, remaining slightly distanced from the main tissue in Fig. 5d, whereas this was not observed for AC. It is likely that this detachment of root cells might be promoted by the microscopy sample preparation method, with the cells more easily dislodged by physical manipulation in *brt-*2 than in AC. Similar root cap cell separation was already described in lines with altered *AtHsfB4* (*SCZ*) function (Begum et al., 2013; ten Hove et al., 2010); this phenotypic similarity in an orthologous gene strongly supports the genetic data that indicates that the S75C mutation in *SolycHsfB4a* is responsible for *brt-2*.

## Discussion

### The *brt-2* mutant shows a perturbed root phenotype leading to delayed shoot development

The *brt-2* mutant is a member of the large monogenic mutant collection of TGRC which contains more than 1000 mutant lines. Among these, *brt-2* is one of only a few lines defined as primary root mutants. Despite normal seed germination, the *brt-2* mutant shows a severe phenotype with bushy, twisted roots during the early stages of seedling development, accompanied by delays in general plant growth. These curling roots lack fine lateral roots, further reducing the overall root system size. Compared to AC, fully established *brt-2* mutant plants were observed to have a greater tendency to wilt in strong sunlight, likely indicating that the *brt-2* root system is unable to meet the demand for water at high transpiration rates. Even though *brt-2* has delayed shoot growth, it is able to undergo normal fruit development and seed set. These fruits usually exhibit cracks which is consistent with intermittent water stress due to poor root functioning; an episode of water stress may lead to reduced extensibility of the epidermis, followed by resumption of pericarp expansion, so creating increased tissue tension and cracking (Khadivi-Khub, 2015).

We used the grafting capability of tomato to investigate whether the causative mutation is primarily acting through changes in the root system, or if it directly influences the scion growth as well. The self and reciprocal grafting between AC and *brt-2* clearly showed that the phenotype is determined by the root genotype, indicating a local effect of the gene altering root development and a secondary effect on the shoot due to impaired root function.

### Genetic mapping revealed *SolycHsfB4a* as candidate gene for *brt-2*

For mapping *brt-2*, we created a segregating F_2_ population crossing the mutant line with a widely used, indeterminate tomato cultivar, Ailsa Craig. Resequencing by NGS of both parental lines identified a relatively low number of DNA polymorphisms on chromosome 4, such that a genotyping-by-sequencing (GBS) approach was not feasible, indicating close similarity between parents. The NGS polymorphisms allowed *brt-2* to be mapped to ~ 2 Mbp on the long arm of chromosome 4, and the low number of polymorphisms was very helpful in this case as only one clear candidate polymorphism was found.

The S75C mutation in *SolycHsfB4a* was a unique allele that displayed 100% co-segregation with the phenotype. No other polymorphisms were detected in the mapping interval that could explain the *brt-2* phenotype, leading to the conclusion that *SolycHsfB4a* was the only candidate and the causative gene for *brt-2*. The physical distance between *SlIAA9* (*entire*) and *SolycHsfB4a* is 1.68 Mbp, consistent with the low recombination frequency previously observed between the two loci (Voland and Zobel, 1988).

### The *brt-2* allele of *SolycHsfB4a* contains a uniquely recessive DNA binding domain variant

The secondary structure of Hsf DNA binding domain (DBD) consists of three-helix bundles enveloped with a four-stranded antiparallel β sheet (Harrison et al., 1994). The order of these structural elements within the DBD is α1-β1-β2-α2-α3-β3-β4 located at the amino terminus of the protein; this pattern is unchanged among the different *Hsf* gene family members. For DNA binding to occur a trimer of Hsf polypeptides is formed.

The S75C *brt-2* mutation is located in the conserved Hsf DBD domain and a blast search revealed that S75 in the sequence context SFVRQ is absolutely conserved across all eukaryotic organisms and in all members of the Hsf family (Lv et al., 2014). A crystal structure for human Hsf1 shows that the equivalent of S75 (S68 in human Hsf1) occurs within the α3 helix that contacts the major groove of the DNA within the specific nucleotide sequence of the heat shock element (HSE), forming a hydrogen bond with the DNA phosphate backbone (Neudegger et al., 2016). The S75C mutation leads to replacement of a single atom (oxygen of serine exchanged for sulphur of cysteine) and would be expected to disrupt the hydrogen bond, likely weakening the binding of BRT-2 protein to DNA. Since the HsfB4a class lacks the transcriptional activator domain of other Hsf proteins, it is believed to repress transcription by binding to and “blocking” the HSE; thus S75C is likely to reduce the repressor activity of SolycHsfB4. Interestingly, in human Hsf4 mutants that cause congenital lamellar cataracts, all known amino acid substitutions located in the DBD are dominant negative mutations because of the formation of dysfunctional heterotrimers in heterozygous cells (Berry et al., 2017; Jiao et al., 2019). However, the *brt-2* mutation is highly unusual in being within the DBD, but also fully recessive. The S within SFVRQ has not been reported to be mutated in human HSFs linked to congenital disease, or in any other natural variants, and thus *brt-2* appears to have a recessive DBD mutation not previously described in the extensive literature on HSFs in many eukaryotic organisms. In SCZ (discussed below), the reported allelic series of loss-of-function mutations are all outside the DBD and recessive (ten Hove et al., 2010). The recessive nature of the S75C mutation in the DBD could indicate that both DNA binding and trimer formation are disrupted, e.g. by a major disruption in protein folding, effectively creating a null mutant. Perhaps a less likely explanation, given the highly conserved motif SFVRQ, is that S75C causes of a gain-of-function of the DBD that is negated by trimerization with wild type polypeptides in the heterozygote, making it recessive. In general, recessive gain-of-function mutations occur rarely (Liu et al., 2020).

### The tomato *SolycHsfB4a* gene is orthologous to Arabidopsis *SCHIZORIZA*

The closest homologue of *SolycHsfB4a* is *At1g46264* in *Arabidopsis*, also known as *SCHIZORIZA* (*SCZ*) or *AtHsfB4* which has its highest expression in root and shoot apices of *A. thaliana* (Winter et al., 2007; Begum et al., 2013). Similarly, in the TomExpress database (Zouine et al., 2017) *SolycHsfB4a* has high expression in root, and leaf and shoot meristematic tissues (Fig. S2, Table S3); it is therefore not root specific despite the clear evidence from reciprocal grafting that the main effect of the mutation acts in the root, evidence that has not been reported before for SCZ where grafting is more technically challenging. This appears to be an example where transcription profile and gene function do not coincide. The CoNekT database (Proost and Mutwil, 2018) shows mRNA levels in different root zones: meristematic, elongation, differentiation, root hairs and bulk root. This data (Fig. S3, Table S4) shows that, within roots, *SolycHsfB4a* is most highly expressed in the meristematic zone (25.8 TPM), with the next highest expression in the elongation zone (5.1 TPM). Similarly, the *AtHsfB4* promoter directs GUS expression to root meristem tissue, and is specific for stele, cortex, endodermis and the quiescent centre (QC) (Begum et al., 2013).

Even though *HsfB4* is a member of the large gene family of heat shock transcription factors (Hsf), heat stress activation is not the unique functional trigger among the rather diverse members (Swindell et al., 2007). *Arabidopsis HsfA6a* and *HsfA6b* have increased expression in samples treated with osmotic, salt, and cold stress, while *HsfB1*, *HsfA2*, *HsfA4a*, *HsfA4c* and *HsfB2a* are induced upon biotic stress (von Koskull-Döring et al., 2007). Seventeen *Hsf* genes were isolated from a wild diploid woodland strawberry and they were induced by various abiotic and biotic stresses (Hu et al., 2015). Arabidopsis has 21, and tomato 24 *Hsf* genes (Tang et al., 2016). Hsf proteins bind to heat shock elements (HSEs) within the promoters of target genes, including heat shock protein (*HSP*) genes that act as molecular chaperones in a wide range of plant responses to abiotic and biotic stimuli and during plant development.

### The mutant of *SolycHsfB4a, brt-2*, has a similar phenotype to Arabidopsis lines with perturbed expression of *SCZ* (*AtHsfB4*)

*SCZ* encodes a nuclear protein regulating cell division asymmetry in *A. thaliana* roots through coordinated action with *SCARECROW* (*SCR*) (Mylona et al., 2002; ten Hove et al., 2010; Pernas et al., 2010): together these two genes direct the development of the root cap, epidermis and ground tissue (cortex and endodermis). The *scz* transposon knock-out mutant shows a perturbed, asymmetric cell division pattern that can be seen in the root meristem from the torpedo embryo stage (Pernas et al., 2010). The knock-out mutant has a range of complex phenotypes: additional and aberrant periclinal cell divisions; subepidermal cell layers that produce root hairs; a disorganised arrangement of thrichoblasts and atrichoblasts; and supernumerary layers of epidermal cells and/or lateral root cap cells that become less distinct and have a tendency to separate from the root as it matures (ten Hove et al., 2010; Mylona et al., 2002; Pernas et al., 2010). This disrupted root function is coupled with a reduced shoot stature (Mylona et al., 2002) and reduced root growth (ten Hove et al., 2010). It was concluded that SCZ acts to restrict epidermal fate to the outer layers of the root, and is required to maintain the stem cells that give rise to cortex and endodermis and for normal QC development (Pernas et al., 2010). Since the QC represses the differentiation of columella stem cells and the *scz* mutant has a defective QC, *scz* also exhibits precocious differentiation of columella stem cells (Pernas et al., 2010).

The above studies on *SCZ* mutants in Arabidopsis have used knock-out mutants rather than amino acid substitution mutants (ten Hove et al., 2010; Mylona et al., 2002; Pernas et al., 2010), thus the S75C mutation in *SolycHsfB4a* might behave differently if it retains some functional aspects of the protein. It is therefore necessary to consider also the phenotypes observed in Arabidopsis *AtHsfB4* overexpression (OE) lines. *AtHsfB4* is normally expressed specifically in ground tissue cells and the QC, so OE of *AtHsfB4* using CaMV 35 s promoter would increase expression in these same tissues, but also ectopically in other root tissues (Begum et al., 2013). The *AtHsfB4-OE* lines showed specific root morphological changes: while the aerial parts of the plant looked normal, there was a clear delay in root growth and development of *HSFB4-OE* lines compared to WT. Microscopic images revealed distinct structural changes at the root surface, thickening of the meristematic region showing a rough surface and a detachment of cells in the elongation and maturation root zones. *HSFB4-OE* lines have additional periclinal divisions which lead to the generation of extra cell layers in the ground tissue (cortex and endodermis) and additional layers of lateral root cap cells were also observed. Thus, the production of extra abnormal layers of cells and cellular detachment is a feature common to both *SCZ* knock-out and over-expression.

The *brt-2* line shows high cell proliferation around the root cap and the meristematic cells (Fig. 5). These extra cells are easily detached from the main root tissue, principally from the root cap columella, lateral root cap and possibly also the cell division zone. These complex features of cell proliferation and detachment are common between *Arabidopsis* SCZ knock-outs, SCZ ectopic overexpression lines, and the *brt-2* mutant, so while this strongly supports *brt-2* as orthologous to SCZ, it is not possible to be sure from the phenotype if the S75C mutation in *brt-2* is causing a complete loss or an alteration of protein function. However, since the *brt-2* mutant allele is recessive, it seems more likely that the brt-2 mutant protein has lost both its HSE binding function (losing its repressor function) and its ability to form trimers, so preventing it from behaving like other DBD mutants which are dominant through the poisoning of trimers in the heterozygote.

### Potential for *BRT-2* alleles to provide root-knot nematode (RKN) resistance

RKN resistance is an essential goal in rootstock breeding to avoid significant crop losses (Okorley et al., 2018). In tomato, it was previously found that silencing *HSFA1a* compromised Mi-1.2-mediated RKN resistance by preventing the hypersensitive response (Zhou et al., 2018). However, the roles of other tomato *HSF* genes in RKN resistance are unknown.

RKNs strongly induce the expression of *SCZ/AtHsfB4* in *Arabidopsis* roots as part of the process leading to organogenesis of root galls, specialised structures that develop in the vascular tissue of roots and provide nourishment to the RKNs (Olmo et al., 2020). Moreover, three loss-of-function *scz* mutants in Arabidopsis showed “a severe decrease in nematode infection and reproduction”, whereas *SCZ* overexpression and loss-of-function of the related genes *AtHSFA1a* and *AtHSFA1b* had no effect, indicating a specific role of *AtHsfB4* loss-of-function in RKN resistance (Olmo et al., 2020). It was suggested that recruitment of the host pathways for root apical meristem generation is part of the mechanism by which RKNs generate galls, and that disruption of these pathways, including by inactivation of *HSFB4*, could provide RKN resistance (Olmo et al., 2020). *SCZ* loss-of-function was associated with an anecdotal reduction in shoot stature; although this has not been quantified in Arabidopsis (Mylona et al., 2002), there is a likely trade-off between root function and RKN resistance.

Here we provide the first description of a tomato HSFB4 mutant (*brt-2*). Although it is highly disruptive to root and shoot growth, the report by Olmo et al. (2020) suggests that this material will be RKN resistant and there remains the possibility that natural or engineered functional or expression variants of *BRT-2*/*SolycHsfB4a* that combine acceptable scion growth rates coupled with RKN resistance could be developed and deployed in tomato rootstock cultivars.

## Methods

### Plant material and growth

Tomato cultivar Ailsa Craig carrying an introgression from *Solanum peruvianum* on chromosome 9 with the resistant allele of the *Tobacco mosaic virus resistance-2* locus (*Tm-2*^*a*^) was used as one parent (AC). This was crossed with the *bushy root-2* (*brt-2*) mutant line (TGRC accession LA3206), generating F_1_ plants that were self-pollinated to produce F_2_ seeds for use as a mapping population. Seed accessions are given in Fig. S1.

Seed were extracted from red ripe tomato fruits and separated seeds and gel were incubated overnight after adding 2–3 volumes of 0.12 M hydrochloric acid (Sigma-Aldrich), 1 g L^− 1^ brewer’s pectolase (Ritchie, Burton-upon-Trent, UK) at room temperature. The seeds were washed thoroughly in tap water and dried at room temperature for at least five days. Before germination, all seeds were sterilised in 0.45% w/v sodium hypochlorite for 30 min and then rinsed in tap water to avoid seed-borne viral transmission. Seed were germinated as described (Silva Ferreira et al., 2018) before transplanting into 8 L pots of Sinclair multipurpose compost (LBS Horticulture, Colne, UK). Pots were irrigated according to demand and were fed twice a week with Hoagland solution (5 mM K_2_SO_4_; 1 mM H_3_PO_4_; 5 mM Ca (NO_3_)_2_; 2 mM MgSO_4;_ 100 μM EDTA Fe-Na; 42.2 μM H_3_BO_3_; 9.1 μM MnCl_2_; 0.76 μM ZnSO_4_ and 0.32 μM CuSO_4_, pH 5.8 adjusted with H_3_PO_4_), at half strength before flowering and full strength after flowering. Four-week-old plants were phenotyped for the *brt-2* trait and young leaf material used for DNA extractions.

### Grafting experiments

Pre-germinated AC and *brt-2* seeds were sown in 24-module standard seed trays with multipurpose compost and were grown in the glasshouse. Three-week-old plants were grafted in all combinations following the Japanese top-grafting method using silicon tube-shaped clips (Rivard and Louws, 2006). After grafting, plants were transferred to a healing chamber shaded from direct sunlight and providing 100% humidity levels via a LT1 Mist-Wean Controller connected to a Wet Leaf electrode (Access Irrigation, Northampton, UK) and two CoolNet Pro-4 fogging heads (Netafim, Hatzerim, Israel); the controller generated 5 s of water misting repeating after a 15 min delay and influenced by a level 2 sensitivity threshold. Plants were weaned from the healing chamber six days after grafting by reducing humidity over three days, and were transplanted into 22 cm diameter, 10 L pots filled with Sinclair multipurpose compost placed on a bench in the glasshouse, and hand watered on demand. Nine-week-old grafted plants were assessed: roots were washed from the compost, and root and shoot dry weights (DW) were measured.

### DNA extraction and KASP genotyping

Genomic DNA extraction from young leaves and the KASP/KBD assays were performed as described (Silva Ferreira et al., 2018). All KBD assays were developed by LGC (Teddington, UK) based on the provided nucleotide polymorphism and flanking sequence data (Table S1). The KASP genotyping results were analysed in CFX96 qPCR machines using the “Allelic Discrimination” feature of CFX manager software (BioRad, Watford, UK).

### NGS genomic data generation and sequence analysis

Genomic DNA from AC and *brt-2* plants was extracted using the DNeasy plant mini kit (Qiagen; Manchester, UK), according to the manufacturer’s instructions. Both lines were subjected to paired-end sequenced using Illumina HiSeq X platforms. The data comprised 389,240,368 (AC) and 431,168,724 (*brt-2*) 100 bp reads representing ~39x and ~ 43x coverage of average read depths, respectively. Data is available from SRA accession PRJNA750735 (NCBI). Reads were aligned to the SL2.50 (Heinz 1706) reference genome and variants were called using the “Alpheus” pipeline (Miller et al., 2008). AC possessed 1,139,329 and *brt-2* 193,743 sequence variants compared to Heinz 1706. The resulting VCF files were loaded into Integrative Genomics Viewer (IGV) to visualise the sequence variations between the parental genomes along the 12 chromosomes (Robinson et al., 2011) and to design KASP markers for the genetic mapping procedure.

For protein comparisons, the open source BoxShade Server (version 3.21, EMBnet) was used with the default values. The BoxShade program used the Multiple Sequence Alignment (MSF) files generated by ClustalW Multiple Alignment feature (with default values) of BioEdit version 7.2.5 (Hall, 1999). The PROVEAN (Protein Variation Effect Analyser) tool was used to predict whether an amino acid substitution would impact on the biological function of a protein (Choi et al., 2012).

### Root microscopy

AC and *brt-2* seeds were germinated as described above. Seeds were placed on moistened filter paper in Petri dishes sealed with Parafilm™ to maintain humidity and covered with foil to exclude light. They were left in a growth room at 22 °C for 7 days. For the microscopic studies, approx. 1 cm lengths of root (with root tip) were removed with a razor blade and placed in water on a cavity slide, a coverslip was mounted on top. Microscopy was carried out with a Leica DM6 B Compound Microscope, “Brightfield” and “Differential Interference Contrast” methods were used. Images were captured using 10x and 20x objectives via a Zeiss Axiocam 506 colour (6 Megapixel) microscope camera. The image acquisition and storage software IMS V18Q4 (Imagic Imaging Ltd), was used to capture conventional single images, and extended depth of field images via the software’s “multifocus live” mode to generate composite images.

### Supplementary Information

**Additional file 1 Fig. S1** Pedigree of the *brt-2* mapping population. WSS numbers are the Cranfield seed accessions used for the study. The percentage of WT and *brt-2* phenotypes in the F_2_ population are indicated

**Additional file 2 Fig. S2** Expression pattern of *Solyc04g078770* in the Tomexpress RNA-seq database. Expression values were normalised by mean counts per base. Detailed expression values are in Table S3.

**Additional file 3 Fig. S3** Expression profile of *Solyc04g078770* in the CoNekT RNA-seq database. Expression values are given in transcripts per kilobase million (TPM), normalised for read count and gene length. Bars represent mean value; circles represent minimum and maximum values. Bars are colour coded: white, roots; green, vegetative shoot; dark grey, callus; light grey, seeds; red, fruit; yellow, floral reproductive tissues.

**Additional file 4 Table S1** Shoot and root dry weight (g) of reciprocal grafted plants. Grafts are described as shoot/root, eg. AC/*brt-2.* SDW, shoot dry weight; RDW, root dry weight. *n* = 8 or 9. This data was used for Fig. 2.

**Additional file 5 Table S2** Sequences submitted to LGC Ltd. for design of KBD assays. SNPs were detected by whole genome sequencing of parental lines (AC and *brt-2*) using SL2.50 tomato reference genome.

**Additional file 6 Table S3** Expression values for *Solyc04g078770* in the RNA-seq database of TomExpress

**Additional file 7 Table S4** Expression values for *Solyc04g078770* in the CoNekT database

## Data Availability

Genome sequence data of the *brt-2* and Ailsa craig^Tm-2a^ line is available from SRA accession “PRJNA750735” in NCBI; https://www.ncbi.nlm.nih.gov/bioproject/PRJNA750735/.

## Abbreviations

AC: Ailsa Craig
DBD: DNA-binding domain
DW: Dry weights
GBS: Genotyping-by-sequencing
HSE: Heat shock element
Hsf: Heat shock transcription factors
HSP: Heat shock protein
IGV: Integrative Genomics Viewer
MSF: Multiple Sequence Alignment
NGS: Next generation sequencing
OE: Overexpression
PROVEAN: Protein Variation Effect Analyser
QC: Quiescent centre
SCR: SCARECROW
SCZ: SCHIZORIZA
TGRC: Tomato Genetics Resource Center
Tm-2a: Tobacco mosaic virus resistance-2 locus
WT: Wild type

## References

[CR1] Aflitos S (2014). Exploring genetic variation in the tomato (Solanum section Lycopersicon) clade by whole-genome sequencing. Plant J.

[CR2] Ahn SG, Liu PCC, Klyachko K, Morimoto RI, Thiele DJ (2001). The loop domain of heat shock transcription factor 1 dictates DNA-binding specificity and responses to heat stress. Genes Dev.

[CR3] Alaguero-Cordovilla A, Belén Sánchez-García A. Sergio Ibáñez, |, Albacete, | Alfonso, Cano, A., Acosta, M., and Manuel Pérez-Pérez, J. (2021). An auxin-mediated regulatory framework for wound-induced adventitious root formation in tomato shoot explants. 2021. 10.1111/pce.14001 33464573

[CR4] Alonge, M. et al. (2020). Major impacts of widespread structural variation on gene expression and crop improvement in tomato. Cell 182: 145-161.e23. 10.1016/j.cell.2020.05.021 PMC7354227 32553272

[CR5] Begum T, Reuter R, Schöffl F (2013). Overexpression of AtHsfB4 induces specific effects on root development of Arabidopsis. Mech Dev.

[CR6] Berry V, Pontikos N, Moore A, Ionides A, Plagnol V, Cheetham ME, Michaelides M (2017). A novel missense mutation in HSF4 causes autosomal-dominant congenital lamellar cataract in a British family. Nat Publ Group.

[CR7] Berz J, Simm S, Schuster S, Scharf KD, Schleiff E, Ebersberger I (2019). Heatster: A database and web server for identification and classification of heat stress transcription factors in plants. Bioinformatics and Biology Insights.

[CR8] Choi Y, Sims GE, Murphy S, Miller JR, Chan AP (2012). Predicting the functional effect of amino acid substitutions and Indels. PLoS One.

[CR9] Clayberg CD, Butler L, Kerr EA, Rick CM, Robinson RW (1966). Third list of known genes in the tomato: with revised linkage map and additional rules. J Hered.

[CR10] Elshire RJ, Glaubitz JC, Sun Q, Poland JA, Kawamoto K, Buckler ES, Mitchell SE (2011). A robust, simple genotyping-by-sequencing (GBS) approach for high diversity species. PLoS One.

[CR11] Gálvez A, del Amor FM, Ros C, López-Marín J (2019). New traits to identify physiological responses induced by different rootstocks after root-knot nematode inoculation (Meloidogyne incognita) in sweet pepper. Crop Prot.

[CR12] Gao L, Gonda I, Sun H, Ma Q, Bao K, Tieman DM, Burzynski-Chang EA, Fish TL, Stromberg KA, Sacks GL, Thannhauser TW, Foolad MR, Diez MJ, Blanca J, Canizares J, Xu Y, van der Knaap E, Huang S, Klee HJ, Giovannoni JJ, Fei Z (2019). The tomato pan-genome uncovers new genes and a rare allele regulating fruit flavor. Nat Genet.

[CR13] Gregory PJ, Atkinson CJ, Bengough AG, Else MA, Fernández-Fernández F, Harrison RJ, Schmidt S (2013). Contributions of roots and rootstocks to sustainable, intensified crop production. J Exp Bot.

[CR14] Hall TA (1999). BioEdit: A user-friendly biological sequence alignment editor and analysis program for windows 95/98/NT. Nucleic Acids Symp Ser.

[CR15] Hammer GL, Dong Z, McLean G, Doherty A, Messina C, Schussler J, Zinselmeier C, Paszkiewicz S, Cooper M (2009). Can changes in canopy and/or root system architecture explain historical maize yield trends in the U.S. corn belt?. Crop Sci.

[CR16] Harrison CJ, Bohm AA, Nelson HCM (1994). Crystal structure of the DNA binding domain of the heat shock transcription factor. Science.

[CR17] Hochmuth GJ (1985). A gene affecting tomato root morphology. HortScience.

[CR18] Hu Y, Han YT, Wei W, Li YJ, Zhang K, Gao YR, et al. Identification, isolation, and expression analysis of heat shock transcription factors in the diploid woodland strawberry Fragaria Vesca. Front Plant Sci. 2015;6. 10.3389/fpls.2015.00736. 10.3389/fpls.2015.00736 PMC4569975 26442049

[CR19] Jiao X, Khan SY, Kaul H, Butt T, Asif Naeem M, Riazuddin S, Fielding Hejtmancik J, Amer RiazuddinID S (2019). Autosomal recessive congenital cataracts linked to HSF4 in a consanguineous Pakistani family. Autosomal recessive congenital cataracts linked to HSF4 in a consanguineous Pakistani family.

[CR20] Kerr, E.A. (1982). Upright growth (up) and aerial roots-2 (aer-2) – two new genes from White beauty. Report of the tomato genetics cooperative 32: 34-undefined.

[CR21] Khadivi-Khub A. Physiological and genetic factors influencing fruit cracking. Acta Physiol Plant. 2015;37(1). 10.1007/s11738-014-1718-2.

[CR22] Kim JE, Oh SK, Lee JH, Lee BM, Jo SH (2014). Genome-wide SNP calling using next generation sequencing data in tomato. Molecules and Cells.

[CR23] Lin T (2014). Genomic analyses provide insights into the history of tomato breeding. Genomic analyses provide insights into the history of tomato breeding.

[CR24] Lindstrom EW (1933). Hereditary radium-induced variations in the tomato. J Hered.

[CR25] Liu J, Wang Y, Cheng Y (2020). The ESCRT-I components VPS28A and VPS28B are essential for auxin-mediated plant development. Plant J.

[CR26] Lv H, Huang C, Zhang J, Liu Z, Zhang Z, Xu H, You Y, Hu J, Li X, Wang W (2014). A novel HSF4 gene mutation causes autosomal-dominant cataracts in a Chinese family. G3: genes. Genomes, Genetics.

[CR27] Martínez-Andújar C, Albacete A, Pérez-Alfocea F (2020). Rootstocks for increasing yield stability and sustainability in vegetable crops. Acta Hortic.

[CR28] Miller NA, Kingsmore SF, Farmer A, Langley RJ, Mudge J, Crow JA, Gonzalez AJ, Schilkey FD, Kim RJ, van Velkinburgh J, May GD, Black CF, Myers MK, Utsey JP, Frost NS, Sugarbaker DJ, Bueno R, Gullans SR, Baxter SM, Day SW, Retzel EF (2008). Management of High-Throughput DNA sequencing projects: Alpheus. Journal of computer science and systems biology.

[CR29] Motte H, Vanneste S, Beeckman T (2019). Molecular and environmental regulation of root development. Annu Rev Plant Biol.

[CR30] Mylona P, Linstead P, Martienssen R, Dolan L (2002). Schizoriza controls an asymmetric cell devision and restricts epidermal identity in the Arabidopsis root. Development.

[CR31] Neudegger T, Verghese J, Hayer-Hartl M, Hartl FU, Bracher A (2016). Structure of human heat-shock transcription factor 1 in complex with DNA. Nat Struct Mol Biol.

[CR32] Nibau C, Gibbs DJ, Coates JC (2008). Branching out in new directions: the control of root architecture by lateral root formation. New Phytol.

[CR33] Oh KC, Ivanchenko MG, White TJ, Lomax TL (2006). The diageotropica gene of tomato encodes a cyclophilin: A novel player in auxin signaling. Planta.

[CR34] Okorley, B.A., Agyeman, C., Amissah, N., and Nyaku, S.T. (2018). Screening selected Solanum plants as potential rootstocks for the Management of Root-Knot Nematodes (Meloidogyne incognita). International journal of agronomy 2018.

[CR35] Olmo R, Cabrera J, Díaz-Manzano FE, Ruiz-Ferrer V, Barcala M, Ishida T, García A, Andrés MF, Ruiz-Lara S, Verdugo I, Pernas M, Fukaki H, del Pozo JC, Moreno-Risueno MÁ, Kyndt T, Gheysen G, Fenoll C, Sawa S, Escobar C (2020). Root-knot nematodes induce gall formation by recruiting developmental pathways of post-embryonic organogenesis and regeneration to promote transient pluripotency. New Phytol.

[CR36] Pernas M, Ryan E, Dolan L (2010). SCHIZORIZA controls tissue system complexity in plants. Curr Biol.

[CR37] Philouze J (1971). A mutant with roots on the stem. Report of the Tomato Genet Cooperative.

[CR38] Pico MB, Thompson AJ, Gisbert C, Yetişir H, Bebeli PJ (2017). Genetic resources for rootstock breeding. Genetic resources for rootstock breeding.

[CR39] Proost S, Mutwil M (2018). CoNekT: an open-source framework for comparative genomic and transcriptomic network analyses. Nucleic Acids Res.

[CR40] Rick CM, Khush GS (1961). X-ray-induced deficiencies of chromosome 11 in the tomato. Genetics.

[CR41] Rivard CL, Louws FJ. Grafting for disease resistance in heirloom tomatoes. In North Carolina Cooperative Extension Service, pp. 2006:1–8.

[CR42] Robinson JT, Thorvaldsdóttir H, Winckler W, Guttman M, Lander ES, Getz G, Mesirov JP (2011). Integrative genomics viewer. Nat Biotechnol.

[CR43] Sánchez-Rodríguez E, Ruiz JM, Ferreres F, Moreno DA (2012). Phenolic profiles of cherry tomatoes as influenced by hydric stress and rootstock technique. Food Chem.

[CR44] Santa-Cruz A, Martinez-Rodriguez MM, Perez-Alfocea F, Romero-Aranda R, Bolarin MC (2002). The rootstock effect on the tomato salinity response depends on the shoot genotype. Plant Sci.

[CR45] Sato S (2012). The tomato genome sequence provides insights into fleshy fruit evolution. Nature.

[CR46] Schwarz D, Öztekin GB, Tüzel Y, Brückner B, Krumbein A (2013). Rootstocks can enhance tomato growth and quality characteristics at low potassium supply. Sci Hortic.

[CR47] Siddiqui MN, León J, Naz AA, Ballvora A (2021). Genetics and genomics of root system variation in adaptation to drought stress in cereal crops. J Exp Bot.

[CR48] Silva Ferreira D, Kevei Z, Kurowski T, de Noronha Fonseca ME, Mohareb F, Boiteux LS, Thompson AJ (2018). BIFURCATE FLOWER TRUSS: A novel locus controlling inflorescence branching in tomato contains a defective MAP kinase gene. J Exp Bot.

[CR49] Stubbe H (1958). Mutanten der Kulturtomate Lycopersicon esculentum Miller Il. Die Kulturpflanze.

[CR50] Stubbe H (1959). Mutanten der Kulturtomate Lycopersicon esculentum Miller III. Die Kulturpflanze.

[CR51] Stubbe H (1964). Mutanten der Kulturtomate Lycopersicon esculentum Miller V. Die Kulturpflanze.

[CR52] Swindell WR, Huebner M, Weber AP (2007). Transcriptional profiling of Arabidopsis heat shock proteins and transcription factors reveals extensive overlap between heat and non-heat stress response pathways. BMC Genomics.

[CR53] Tang R, Zhu W, Song X, Lin X, Cai J, Wang M, Yang Q (2016). Genome-wide identification and function analyses of heat shock transcription factors in potato. Front Plant Sci.

[CR54] ten Hove CA, Willemsen V, de Vries WJ, van Dijken A, Scheres B, Heidstra R (2010). SCHIZORIZA encodes a nuclear factor regulating asymmetry of stem cell divisions in the Arabidopsis root. Curr Biol.

[CR55] Thompson AJ, Pico MB, Yetişir H, Cohen R, Bebeli PJ (2017). Rootstock breeding: current practices and future technologies. Rootstock breeding: current practices and future technologies.

[CR56] Voland, M.L. and Zobel, R.W. (1988). A morphologic and genetic characterization of two tomato root mutants. Report of the tomato genetics cooperative 38: 47 .

[CR57] von Koskull-Döring P, Scharf KD, Nover L (2007). The diversity of plant heat stress transcription factors. Trends Plant Sci.

[CR58] Winter D, Vinegar B, Nahal H, Ammar R, Wilson G v, Provart NJ (2007). An “electronic fluorescent pictograph” browser for exploring and analyzing large-scale biological data sets. PLoS One.

[CR59] Yu, S. -a. and Yeager, A.F. (1960). Ten heritable mutations found in the tomato following irradiation with X-rays and thermal neutrons. Proceedings. Am Soc Horticult Sci.

[CR60] Zhang J, Chen R, Xiao J, Qian C, Wang T, Li H, Ouyang B, Ye Z (2007). A single-base deletion mutation in SlIAA9 gene causes tomato (Solanum lycopersicum) entire mutant. J Plant Res.

[CR61] Zhou J, Xu XC, Cao JJ, Yin LL, Xia XJ, Shi K, Zhou YH, Yu JQ (2018). Heat shock factor HsfA1a is essential for R gene-mediated nematode resistance and triggers H2O2 production. Plant Physiol.

[CR62] Zobel RW (1972). Genetics and physiology of two root mutants in tomato.

[CR63] Zouine M, Maza E, Djari A, Lauvernier M, Frasse P, Smouni A, Pirrello J, Bouzayen M (2017). TomExpress, a unified tomato RNA-Seq platform for visualization of expression data, clustering and correlation networks. Plant J.

