# Supplementary figures and images for: Missense mutation of a class B heat shock factor is responsible for the tomato bushy root-2 phenotype
Source: Mol Hortic. 2022 Feb 8;2:4. doi: 10.1186/s43897-022-00025-0 (PMC10515254; doi:10.1186/s43897-022-00025-0)

## Slide 1
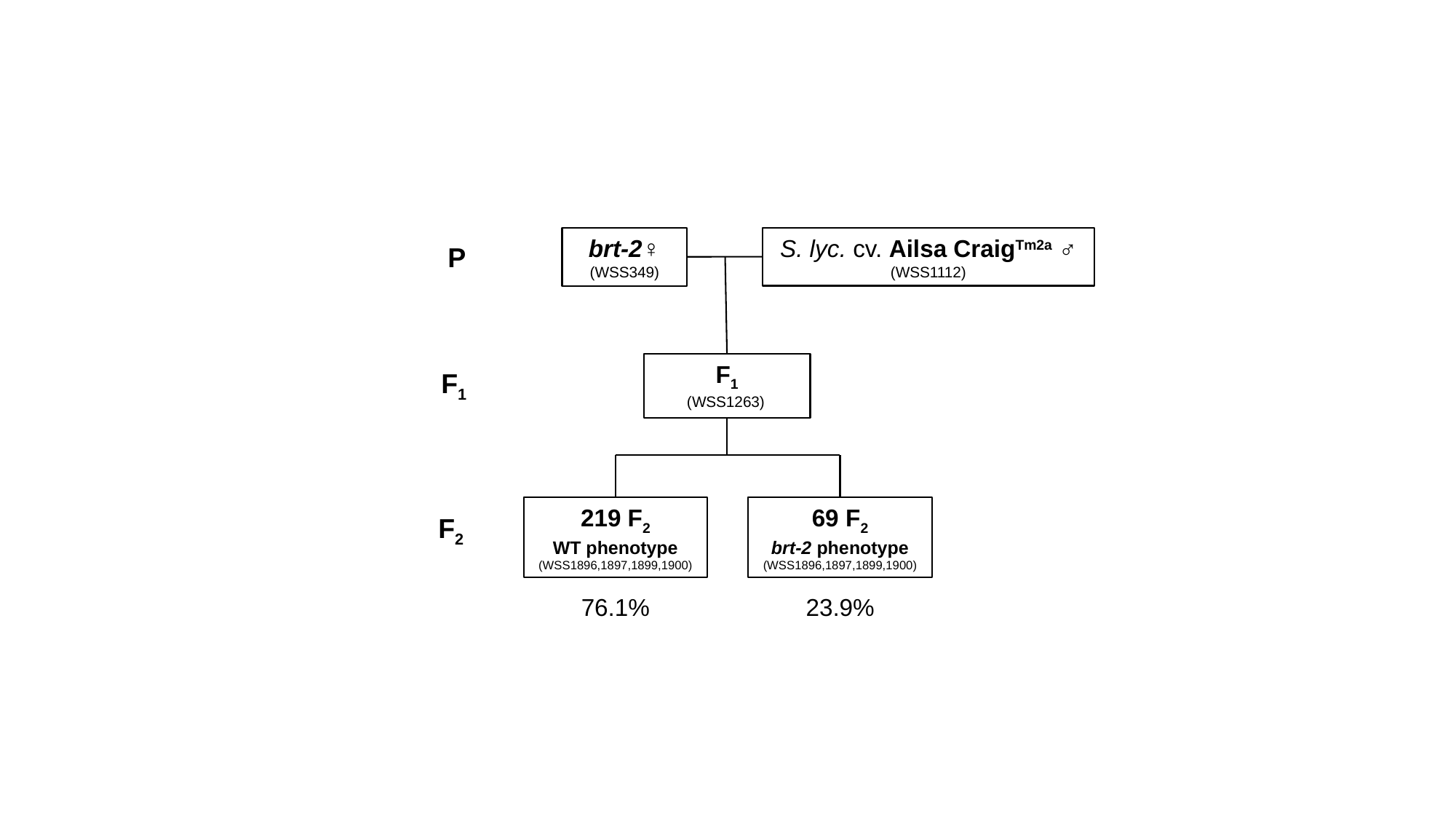

S. lyc. cv. Ailsa CraigTm2a ♂
(WSS1112)
brt-2♀
(WSS349)
P
F1
(WSS1263)
F1
219 F2
WT phenotype
(WSS1896,1897,1899,1900)
69 F2
brt-2 phenotype
(WSS1896,1897,1899,1900)
F2
76.1%
23.9%

Supplement: Supplementary file 1 — Additional file 1 Fig. S1 Pedigree of the brt-2 mapping population. WSS numbers are the Cranfield seed accessions used for the study. The percentage of WT and brt-2 phenotypes in the F2 population are indicated [file 43897_2022_25_MOESM1_ESM.pptx]

## Slide 1
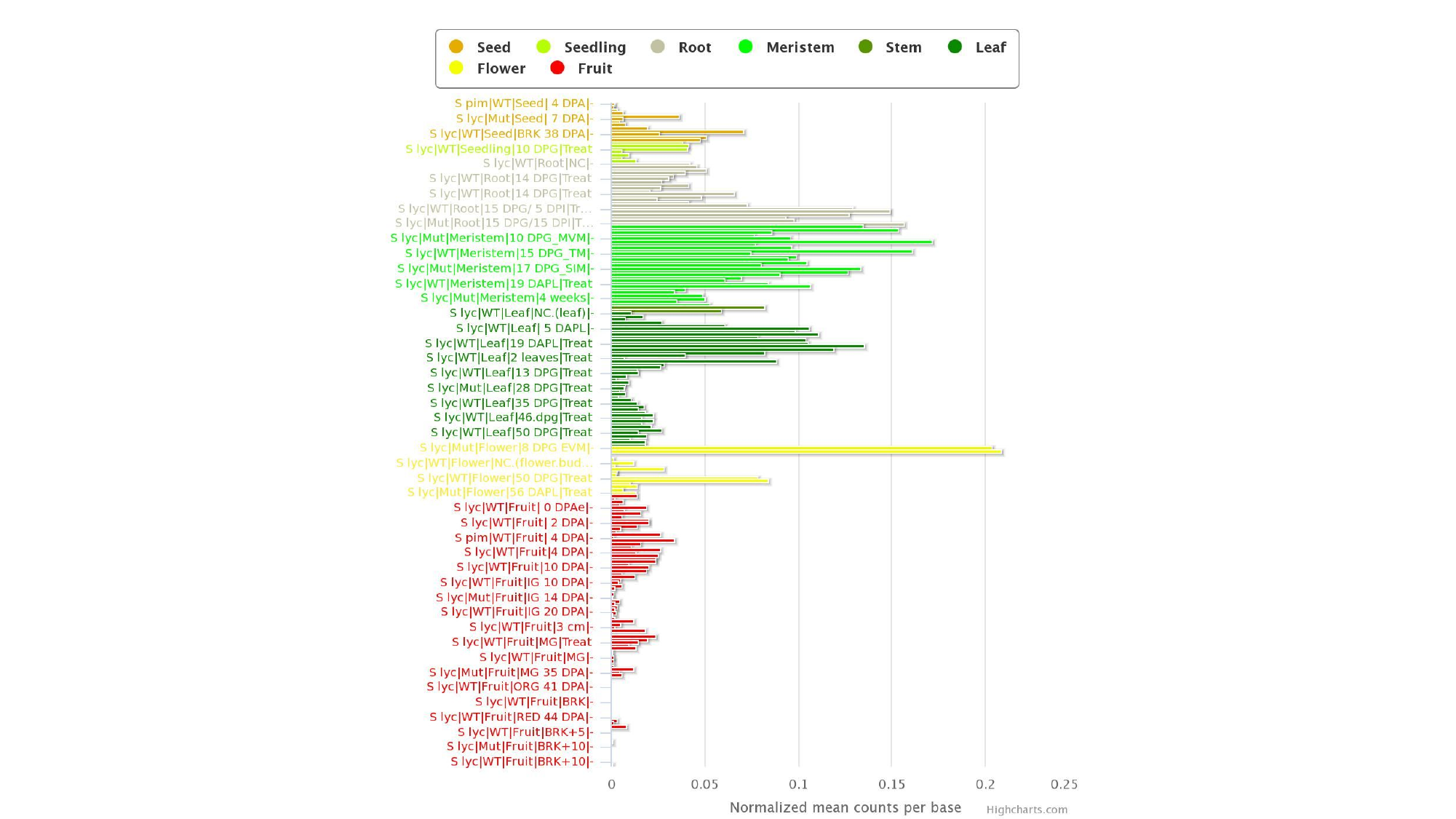

Supplement: Supplementary file 2 — Additional file 2 Fig. S2 Expression pattern of Solyc04g078770 in the Tomexpress RNA-seq database. Expression values were normalised by mean counts per base. Detailed expression values are in Table S3. [file 43897_2022_25_MOESM2_ESM.pptx]

## Slide 1
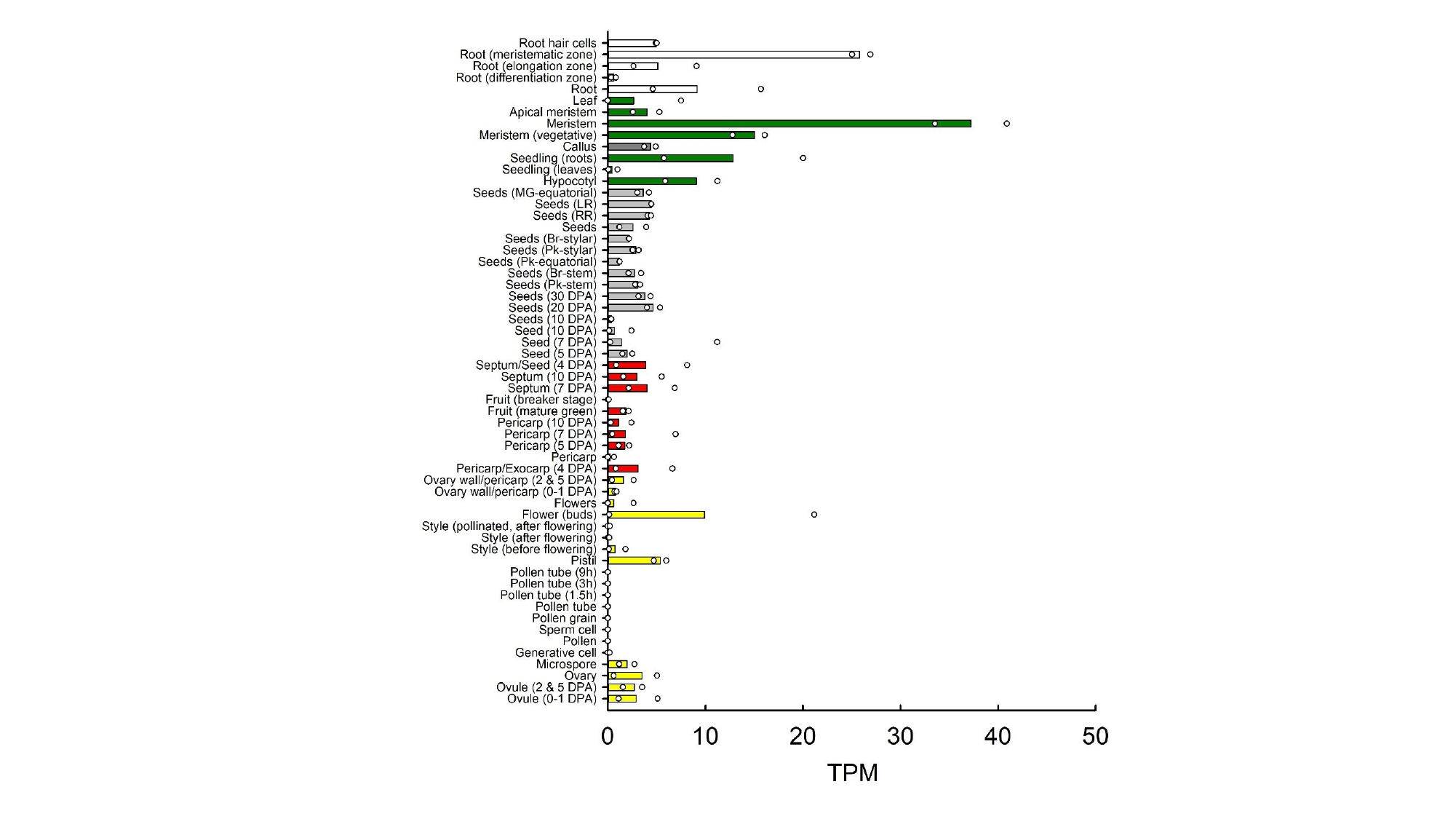

Supplement: Supplementary file 3 — Additional file 3 Fig. S3 Expression profile of Solyc04g078770 in the CoNekT RNA-seq database. Expression values are given in transcripts per kilobase million (TPM), normalised for read count and gene length. Bars represent mean value; circles represent minimum and maximum values. Bars are colour coded: white, roots; green, vegetative shoot; dark grey, callus; light grey, seeds; red, fruit; yellow, floral reproductive tissues. [file 43897_2022_25_MOESM3_ESM.pptx]
